# Application of Impedance-Based Techniques in Hepatology Research

**DOI:** 10.3390/jcm9010050

**Published:** 2019-12-24

**Authors:** Katie Morgan, Wesam Gamal, Kay Samuel, Steven D. Morley, Peter C. Hayes, Pierre Bagnaninchi, John N. Plevris

**Affiliations:** 1 The University of Edinburgh Hepatology Laboratory, Division of Heath Sciences, University of Edinburgh Medical School, Chancellor’s Building, Edinburgh BioQuarter, 49 Little France Crescent, Edinburgh EH16 4SB, UK; Steve.morley@ed.ac.uk (S.D.M.); P.hayes@ed.ac.uk (P.C.H.); J.Plevris@ed.ac.uk (J.N.P.); 2 James Nasmyth Building, Institute of Mechanical, Process and Energy Engineering, Heriot-Watt University School of Engineering and Physical Sciences, Edinburgh EH14 4AS, UK; wesam521@yahoo.com; 3 The Jack Copland Centre, Advanced Therapeutics, Scottish National Blood Transfusion Service, 52 Research Avenue North, Edinburgh EH14 4BE, UK; K.Samuel@ed.ac.uk; 4 MRC Centre for Regenerative Medicine 5 Little France Drive, Edinburgh EH16 4UU, UK; pierre.bagnaninchi@ed.ac.uk

**Keywords:** impedance based cellular assay, ECIS, xCELLigence, TEER, liver, hepatology

## Abstract

There are a variety of end-point assays and techniques available to monitor hepatic cell cultures and study toxicity within in vitro models. These commonly focus on one aspect of cell metabolism and are often destructive to cells. Impedance-based cellular assays (IBCAs) assess biological functions of cell populations in real-time by measuring electrical impedance, which is the resistance to alternating current caused by the dielectric properties of proliferating of cells. While the uses of IBCA have been widely reported for a number of tissues, specific uses in the study of hepatic cell cultures have not been reported to date. IBCA monitors cellular behaviour throughout experimentation non-invasively without labelling or damage to cell cultures. The data extrapolated from IBCA can be correlated to biological events happening within the cell and therefore may inform drug toxicity studies or other applications within hepatic research. Because tight junctions comprise the blood/biliary barrier in hepatocytes, there are major consequences when these junctions are disrupted, as many pathologies centre around the bile canaliculi and flow of bile out of the liver. The application of IBCA in hepatology provides a unique opportunity to assess cellular polarity and patency of tight junctions, vital to maintaining normal hepatic function. Here, we describe how IBCAs have been applied to measuring the effect of viral infection, drug toxicity/IC50, cholangiopathies, cancer metastasis and monitoring of the gut-liver axis. We also highlight key areas of research where IBCAs could be used in future applications within the field of hepatology.

## 1. Introduction

In vitro hepatic studies are commonly used in drug development, toxicity and in liver disease to replace or reduce the number of animals used in experimentation.

The development of organ-on-a-chip technology, which utilises human cells, has the advantages of replacing animal experimentation and providing data more applicable to human hepatic function. However, the assessment of samples is often end-point and destructive to cells. Development of non-invasive label-free methods of monitoring such systems in real-time is desirable and may inform pathways of disease and define targets for pharmacological intervention [1]. One such technique involves monitoring cellular impedance. The advantages of impedance lie in the ability to monitor the cell culture without labels so that there is no interference from foreign molecules or dyes which can alter or influence the target being studied [1,2]. Impedance measurements are also recorded in real-time. The data are recorded in increments which can be sampled several times a second for as long as desired. This is beneficial in pinpointing the time of change in the culture and can be useful in determining the starting point of a toxic effect and a therapeutic window [1,2].

Principles behind this technique are under the larger heading of electrochemical impedance spectroscopy (EIS). EIS is a technique used to determine dielectric properties of a material by interrogating the response of an electrochemical system generated against an AC current [3]. Any perturbation of the signal can be measured in real time as a change in impedance. This technique has been used since the late 19th century [1] for various applications but lends itself to the study of biological material, as cells have electrochemical properties alongside an insulating bi-lipid membrane which impedes the electrical current [2]. 

Impedance-based cellular assays (IBCAs) have emerged as a specific EIS technique which is a non-invasive way to measure the impedance of cells under experimental conditions in real time. Use of IBCAs have been widely reported for a number of tissues, such as cardiomyocytes, neuronal cells, astrocytes, vascular endothelial cells and stem cells [4,5,6,7,8]. Impedance monitoring of cells in vitro relies on cells being cultured directly on microelectrodes or using electrodes in culture media and measurements are recorded as a graph of impedance over time. Depending on the system used, the changes in impedance of cultured cells, in response to various stimuli as compared to untreated controls, can be correlated with alterations in basolateral adhesion, membrane integrity, tight junctions and barrier function.

Within the field of hepatology, membrane integrity and development of cell polarity and tight junctions are all important to the function of the organ as a whole [9]. Therefore, the disruption of these structures can result in hepatic pathologies. Current methods of investigating polarity and tight junction formation, such as immunohistochemistry, protein quantification or gene expression, are usually end-point and destroy cell cultures in the process or require replicate cultures for sampling at different time points. Methods to monitor membrane integrity and tight junctions throughout experimentation would give more complete information on the initiation and progress of disease processes and could also inform drug toxicity studies [10].

IBCAs have been used on a wide variety of biological platforms and tissues, but specific use in hepatic cell culture is not as widely reported. IBCAs could play a major role in the understanding of hepatic and cholestatic disease, giving insight as to the mechanism of toxicity in drug development studies by monitoring barrier function, tight junctions, basolateral adhesion and overall membrane integrity in real-time. 

This review focuses on the uses of IBCAs in hepatology and the need for increasing application of impedance based in vitro assays in this field. We examine various impedance models and how their function and sensitivity has been uniquely used with human hepatic cultures to monitor pathologies and determine changes in aspects of membrane integrity which are vital to maintain hepatic function. 

### 1.1. Common Methods of IBCA in Hepatology Research

**Figure 1:**
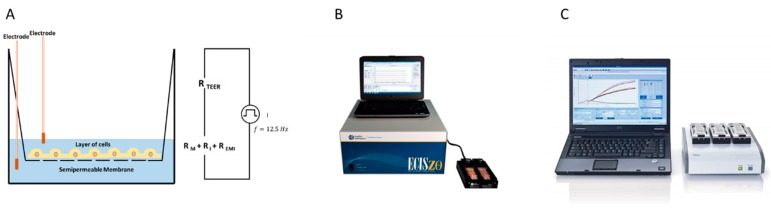
Examples of impedance-based cell assay systems: (**A**) Schematic of transepithelial/endothelial electrical resistance (TEER). TEER measures electrical resistance across a cellular monolayer to confirm the integrity and permeability properties of the membrane. (**B**) Electric Cell-substrate Impedance Sensing (ECIS) Zθ (theta)^TM^ is an automated, non-invasive and label-free instrument capable of monitoring cell behaviour in real-time. The ECIS system is capable of measuring the impedance spectrum, deconvolving data into measurements of total membrane integrity, tight junctions and adhesion, as well as providing a platform for wound healing assays. Courtesy: Applied Biophysics https://www.biophysics.com/ztheta.php (**C**) xCELLigence^TM^ Example of one of the xCELLigence real time cell analysis systems for use in IBCAs. Courtesy: ACEA Biosciences Inc. https://www.aceabio.com/products/xcelligence-rtca/. The xCelligence and TEER systems monitor total impedance, unlike the ECIS system, which deconvolves data into specific parameters using multiple frequency readouts.

There are several platforms available based on the IBCA concept which apply impedance sensing to monitor changes in cell culture. Here we will highlight some of these platforms most commonly used in hepatology including ECIS^TM^, xCELLigence^TM^ and TEER before discussing their application in the field of hepatology research (Figure 1).

### 1.2. ECIS

**Figure 2:**
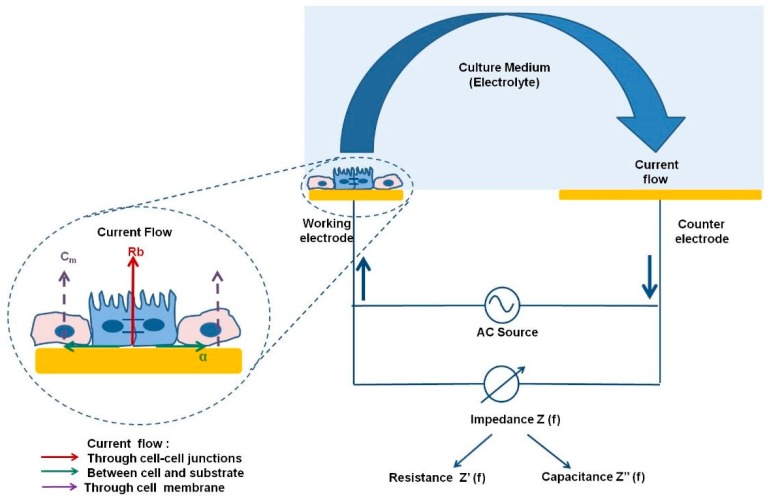
Schematic representation of current flow from ECIS Z theta system; large scale representation of AC current flow within the ECIS theta system with magnification of cells grown on electrode. Cells are grown on working electrodes through which AC current flows under working electrode. AC current can flow under cells showing adhesion (α), through cell membrane showing capacitance (Cm), and between tight junctions of cells (Rb). Alterations in current can give an indication of these three parameters of cellular health. (Figure reproduced with permission from the author Gamal, W., 2015.) [2].

The ECIS model, first reported by Nobel prize laureate Ivor Giaever and Charles Keese, is a real-time, non-invasive, label-free method of monitoring cell behaviour throughout the course of an experiment [11]. Within the ECIS model, cells are grown on a gold electrode through which a low alternating current (AC) at a low voltage produces a small non-invasive current of 1 microamp. Impedance is the measurement of the resistance to this alternating electrical current as it meets opposition, caused in this case by the cell membrane acting as an insulator [11]. As cells grow to confluency across the electrode, impedance of the low AC current increases. The current must therefore go under, around or between cells (Figure 2).

**Figure 3:**
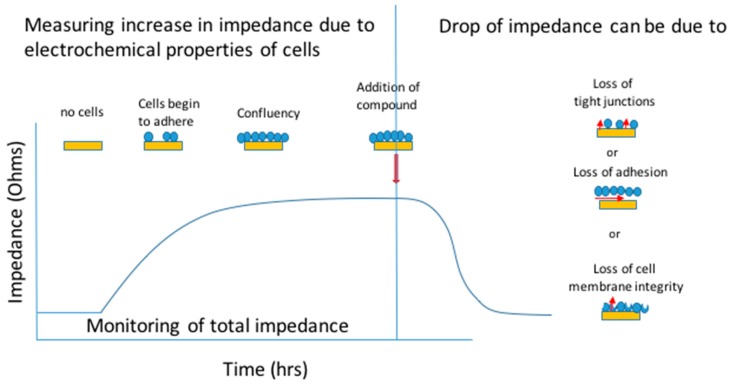
Example of impedance-based measurements with ECIS Zθ; illustration of impedance over time correlated to cell growth on a gold electrode. When no cells are present, there is no impedance to the current. As cells spread, impedance rises, creating a plateau when confluent. During experimentation, using multi-frequency measurements, loss of impedance can be attributed to one of three parameters; loss of cell-cell tight junctions, loss of adhesion or loss of membrane integrity.

ECIS also provides a built-in mathematical modelling of impedance, whereby it is possible to render a measurement of cellular confluence and detect motion on the ECIS gold electrode array [11]. Culturing and monitoring cells on the electrode before the initiation of experimental conditions—for example, the addition of a substance or introducing a wounding assay—acts as an internal control to evaluate changes in total impedance caused by treatment in real time. The measurement of total impedance in Ohms can then be deconvolved to give a prediction of the flow of current between cells (tight junctions/Rb), under cells (basolateral adhesion/alpha) and through cytoplasm (membrane capacitance/Cm) (Figure 3) [11].

Impedance measurements have been used in a variety of ways and is a very simple indicator of confluence and health of a cell culture. Any variance to the culture which causes injury or death to the cells is immediately visible as a decrease in impedance.

### 1.3. xCELLigence™

xCELLigence™ Real-Time Cell Analysis (RTCA) is another commercially designed assay system which uses a non-invasive electrical signal to monitor cellular impedance. It has been applied for assessing cell adhesion, spreading and invasion [12], barrier function [13], cytotoxicity [14], cell signaling and co-culture studies [15,16]. Cells are cultured on gold interdigitated electrodes on the bottom of an xCELLigence™ cell culture well. The cell culture plates are attached to a station, connected to a computer to produce real-time read outs presented as a cell index (CI) defined as the relative change in impedance between the cell-free and cell-covered electrodes at 10, 25 and 50 kHz [14]. This measure of CI is then plotted on a graph of total impedance over time to generate the final results. Unlike ECIS measurements, which can deconvolute data into biological parameters, xCelligence readouts measure only the relative total impedance values. However, the use of appropriate controls and good experimental design make it an extremely flexible and focused platform capable of interrogating chemotaxis, receptor signalling, cell migration/invasion, phenotypic changes caused by various drugs, and prediction of mechanism of action. 

One of the benefits of using ECIS and xCELLigence™ systems is their use of culture plates monitored independently of one another, so several replicates can be run in parallel, allowing multiplexing of quantitative measurements. 

### 1.4. TEER

While ECIS and xCELLigence systems culture cells directly on microelectrodes, transepithelial electrical resistance (TEER) uses two electrodes [17], where an alternating current (AC) is applied across electrodes located at the apical and basolateral membranes. Ohm’s law is used to calculate the changing current between electrodes from real-time measurements, which can be related to the proliferation of cell culture or disruption of cell–cell junctions and which can be applied to investigating biological consequences of toxicity. For instance, the integrity and permeability of the culture can be deduced from TEER measurements [18].

## 2. Importance of Monitoring Tight/Gap Junctions and Polarity in Hepatic Cells

Within the liver, tight junctions between parenchymal cells (hepatocytes and cholangiocytes) are pivotal to maintaining polarity and function of the whole organ [19]. Tight junctions, comprised of various protein complexes, are found at the apices of lateral cell membranes [20]. Bile produced by hepatocytes is secreted into bile canaliculi and then transported to the gall bladder. The number of tight junction strands around bile canaliculi is high [21,22]. They seal bile canaliculi, are selectively permeable, and prevent leakage of water and solutes. As tight junctions are part of the blood–bile barrier, IBCA can provide an indirect measure of the porosity of this system.

Gap junctions make up 3% of the total surface area of a hepatocyte [22], directly regulate intercellular communications and are thought to coordinate bile flow through the acinus by direct communication with the occludin and claudin proteins of the tight junction complex [23]. Due to their integral role with tight junctions, gap junctions are important in cellular polarity and may play a role in signal transfer, proliferation and metabolic signalling along hepatic acini [24].

Changes in membrane or tight junction proteins can be indicative of increased liver pathology as compared to other organs. Mutations in proteins which make up tight junctions are associated with liver diseases such as neonatal ichthyosis, familial hypercholanaemia and sclerosing cholangitis [25]. Also, certain liver diseases, such as primary liver cancer and cholangiocarcinoma, alter protein expression within tight junctions. The level of expression of claudin proteins within the tight junction complex can help in differential diagnosis between hepatic and cholangio carcinomas [26].

## 3. Role of Impedance Based Cellular Assays in the Investigation of Hepatic Disease

### 3.1. Viral Hepatitis 

Viruses such as hepatitis B and C utilise proteins of the tight junction complex as co-receptors for entry into cells [27]. Therefore, compromised membrane integrity is of specific interest and should be monitored in pathologies such as hepatitis C, a leading cause of hepatocellular carcinoma [25].

One challenge to studying hepatitis B and C infection and antiviral therapeutics in vitro is the inability to transfect most cell lines with viruses. Two commonly used hepatic cell lines, Huh-7 and HepaRGs, have both been used extensively in the study of hepatitis B and C [28,29,30,31,32,33,34]. Huh-7 cells, established in 1982 from a human hepatocellular carcinoma [35], are known to produce the human plasma proteins albumin, transferrin, alpha fetoprotein and fibronectin [36]. Their ease of transfectability and their high susceptibility to hepatitis C as they promote the replication of the virus have made the development of drugs possible [30,35,37].

To the best of our knowledge, no literature has been produced to date showing the use of IBCA in the study of hepatitis infection. However, Pennington and Van de Walle [38] used the ECIS Zθ to show proof of the concept that viral infection can be studied using IBCAs. Their study centred around the feline herpes virus type 1 (FHV-1) in kidney cells and detected dose-dependent changes due to virus-induced cell death [39]. 

Further work with Huh-7 or HepaRG cells using IBCA may aid future in vitro studies of viral pathogens and possible therapeutic interventions within hepatology.

### 3.2. Drug Induced Liver Injury

There is a need for non-invasive label-free real-time read outs in drug development, as labels often mask or interfere with the molecule of interest. There are some techniques for monitoring cultures label-free, but combining this with a real-time platform can also identify the time kinetics of the toxic effect and could aid in defining dosing regimens.

Here we will discuss the advantages of IBCA alongside existing assays in a variety of cell lines. 

Failure to detect early hepatotoxicity is a major cause of drug attrition. A study by Gerets et al. [38] used the IBCA xCELLigence™ system together with other techniques to better characterise current hepatic in vitro toxicity models using HepG2, HepaRG and PHH. The xCELLigence™ Real-Time Cell Analysis (RTCA) system has been applied in studies of cell adhesion, spreading and invasion [12], barrier function [13], cytotoxicity [14], cell signalling and co-culture studies [15,16] and more.

Gerets et al. [38] investigated the expression levels of cytochrome P450 (CYP) activity indicative of toxicity using qRTPCR and data generated by the xCELLigence^TM^ system for any predictive correlation with hepatotoxicity. Using this system, they reported that HepG2 cells responded poorly to CYP450 inducers, which correlated with gene expression and CYP enzyme activity [38]. CYP was inducible under all circumstances in HepaRG and PHH; again, gene expression and CYP activity correlated [38]. Using standard assays and comparing with LC50 cytotoxicity curves generated from xCELLigence^TM^ data, they showed that all cell lines were sensitive to hepatotoxins, though none at a high enough level to use in predictive experiments [38]. It is important to note here that a loss of impedance does not necessarily mean a loss of cell viability [3,40,41], but detects a change in the membrane.

In a further study from this group [42], the effect of 5-day exposure to 50 compounds on the viability of HepG2, HepaRG, PHH and primary rat hepatocytes was investigated by comparing the cell index (CI) generated from xCELLigence monitoring with viability measurements based on proteases present in culture using the Promega CellTiter-Fluor assay. The viability data from the two platforms showed a correlation for HepG2 and HepaRGs, but not for PHH or primary rat hepatocytes. Other studies highlight the limitations of poor correlation between in vitro rat and human hepatocyte models [43].

There is currently a lack of comparison of impedance vs. classical toxicity assays. 

Atienzar et al. [42] also investigated the application of xCELLigence^TM^ specific real-time cell analysis to monitor the effect of calcium modulators, anti-mitotics and DNA damaging agents. They determined that it may be possible to identify specific impedance-based signatures dependent on the mechanism of action, although further work needs to be done. These studies showed an important correlation that directly relates impedance measurements to biological viability and activity.

Impedance-based assays combined with MTT, total ATP or other classical viability and metabolic assays may be important for elucidating the mechanism of drug action at a cellular level. These studies highlight the benefits of using a sensitive real-time model for DILI and the generation of predictive values, such as LC50, alongside traditional viability assays, predictability studies and gene expression data. It also highlights the benefit of seeing in real time when damage or change occurs in the cell. This information is useful in identifying the time and mechanism of the toxic effect, planning dosing regimens, and also in planning future animal experimentation. Ultimately, knowing how and when to expect an effect could reduce the number of animals needed in drug toxicity testing. 

### 3.3. Toxicity Assays Using HepaRG Cells

The HepaRG™ cell line is a human line that has emerged in recent years as a suitable alternative to other immortalized cell lines for toxicology studies, with function comparable to PHH [2]. The HepaRG™ cell line was isolated from a female around age 30 with grade I differentiated hepatocellular carcinoma concurrent to hepatitis C [44]. These cells are unique in that they are a bipotent line, which can be differentiated to generate an intrinsic co-culture of hepatocytes and cholangiocyte-like cells, giving a better representation of the liver parenchyma.

Spectral karyotyping of HepaRGs™ revealed a stable and unique genotype which is consistently maintained through several passages. The cultures remained stable for 4 weeks and beyond, and retained characteristics of differentiation, making HepaRG™ cells a viable model for drug toxicity testing. In contrast to other hepatic cell lines, fully differentiated HepaRGs™ provide a unique co-culture of cholagiocyte-like and hepatocyte cells, making them a useful model for drug transporter studies and for use in cholestatic models [40,43,45,46].

**Figure 4:**
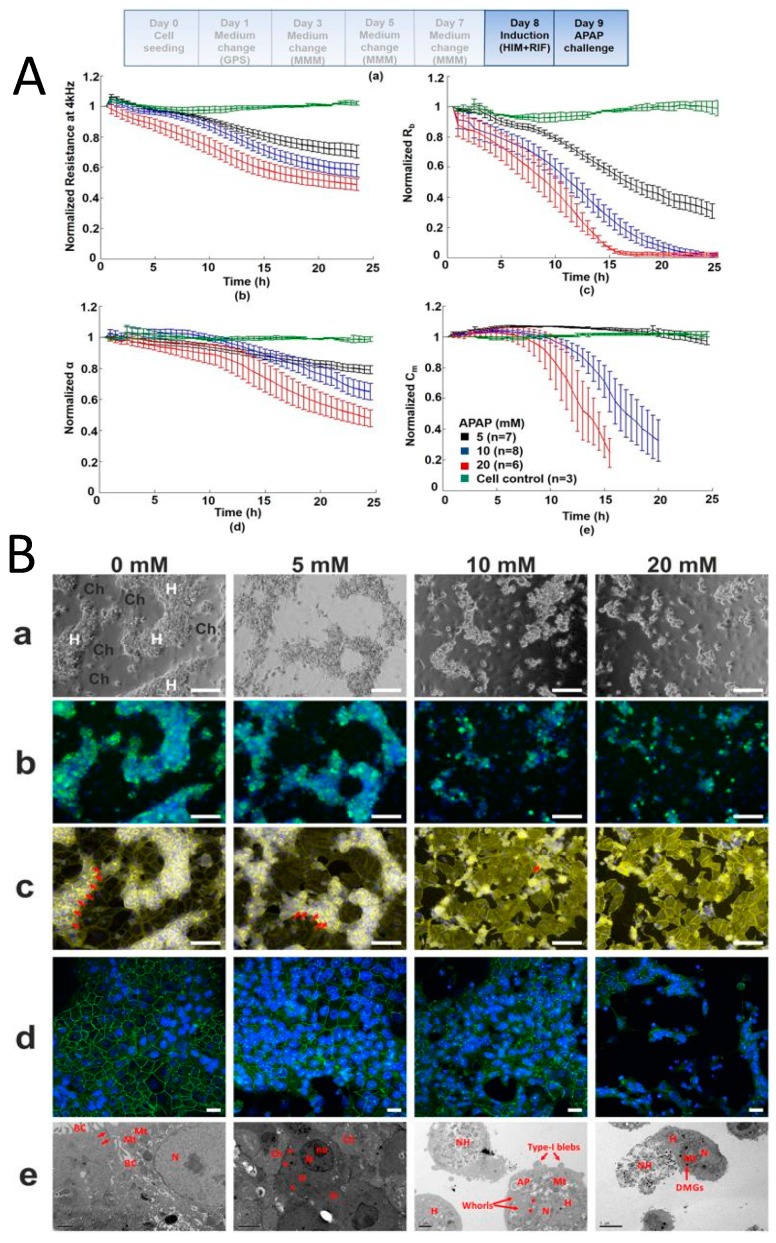
Direct hepatotoxicty signature of HepaRG cells treated with paracetamol; correlation between cellular impedance and immunocytostaining (**A**) HepaRG cells treated for 24 h with paracetamol: untreated cell control; 5 mM; 10 mM; and 20 mM. Paracetamol caused a dose-dependent decline in normalized resistance—a global indicator of cellular status. Graphs show (top left) deconvolution of total impedance, (top right) tight junctions, (bottom left) basolateral adhesion and (bottom right) membrane capacitance (**B**) Correlation to IBCA of confocal microscopy and TEM showing HepaRG cells’ progressive loss of tight junctions with increasing paracetamol concentration. (**a**) phase contrast microscopy where H refer to hepatocytes and Ch to cholangiocytes (**b**) transferrin and Hoescht staining showing loss of function due to destruction of tight junctions (**c**) F-actin staining (yellow) shows breakdown of cytoskeleton with increasing paracetamol concentration. Red arrows show tight junction associated F-actin bands (**d**) confocal microscopy showing loss of tight junction protein ZO-1 (green) (blue nuclear staining with Hoescht) (**e**) representative TEM confirming loss of tight junctions and cells showing a necrotic/apoptotic appearance. Red arrows represent mitochondria and tight junctions at 0mM, an electron dense perimeter around hepatocytes at 5 mM, type I blebbing at 10 mM and dense mitochondrial granules at 20 mM (Reprinted from Gamal et al., 2017) [2].

Published work from our laboratory, measuring total impedance using ECIS technology, has shown a dose-dependent response in fully differentiated HepaRG^TM^ cells following exposure to the model hepatotoxin paracetamol (APAP) [2] (Figure 4). The deconvolution of data revealed a mechanism of dose dependant APAP toxicity not previously described [2]. This is relevant to the clinical presentation of paracetamol toxicity and may inform future investigation into specific molecular targets in the development of therapeutics.

### 3.4. Toxicity Assays Using Pluripotent Stem Cells 

The use of human embryonic stems cells (hESC) and human induced hepatocyte-like cells derived from pluripotent stem cells (hIPS) differentiation to hepatocyte-like cells is very attractive in hepatology research. ECIS offers the advantages of providing hepatocytes from non-diseased tissue, reproducibility between experiments and IPS cells offer the opportunity to study specific disease states and genotypes known to be important in some adverse responses to drugs. Although they have already proven useful in a number of studies, further work is needed to optimise differentiation protocols to achieve mature hepatocyte phenotype and function. hESCs and hIPS could potentially generate tailored models of human liver disease and population-relevant models for drug toxicity testing [47,48,49]. They may also be of particular benefit in the assessment of drugs that cause idiosyncratic toxicity due to genetic polymorphisms.

Zhou et al. [50] showed that impedance data acquired using ECIS to monitor real-time growth kinetics and the differentiation of pluripotent stem cells to hepatic progenitors and hepatocyte-like cells followed established signatures of cell differentiation assessed by microscopy and were measurable in real time by ECIS. The readings from the independent wells were uniform and remained in close proximity throughout the differentiation process [50]. This result is important, as it demonstrates the reproducibility of differentiation between replicates. 

They also used ECIS and Promega CellTiterGlo total ATP assay in parallel to assess the toxicity of compounds BMS4 and BMS5 on hESCs [50]. Using the ECIS Z theta system at a single frequency with one electrode per well showed that there was good correlation of results from the ECIS and CellTiterGlo assays. This demonstrates that through using standard methods, e.g., microscopy, gene expression, functional assays, to support impedance data, it is possible to establish an impedance signal for a particular cell culture under a particular set of conditions. 

This study highlights two very important aspects of IBCAs; namely, their ability to show real-time differentiation and cellular growth kinetics between replicates within an experiment, and substantiating the viability of cells in toxicity testing assays [50]. 

It also demonstrates where the electrode configuration and applied frequency can be chosen according to the study under investigation. Using one electrode per well detects changes within a specific location of cell culture, averaging the signal only from cells growing on that electrode, useful for detecting micromotion of cells or in wound healing assays. However, assays using wells with interdigitated multi-electrode plates give a representation of the average electrical signal across various locations within the well, which is better suited to toxicological studies. 

## 4. Cholestatic Syndromes

### 4.1. Primary Sclerosing Cholangitis

Tabibian et al., 2014 [51] used TEER alongside biochemical and molecular methods to characterise patient-derived cholangiocytes in a study of primary sclerosing cholangitis (PSC). This condition affects biliary epithelial cells, causing inflammation, cholestasis and contributes to end-stage cirrhosis. There are currently no effective therapeutic strategies apart from transplant, which itself carries risk. Due to its complex nature, there are no in vitro models which accurately reflect this disease. As such, Tabibian et al., 2014 [51] aimed to establish a high yield of cholangiocytes from explanted livers of patients with PCS for use in in vitro application. Within their study, they used TEER to measure the conductance through a monolayer, and extrapolated the data to assess the integrity and permeability of the culture, providing an opportunity to explore the biological mechanisms of toxicity, or, in this case, the ability of PSC-derived cholangiocytes to form tight junctions in the culture. PSC patient-derived cholangiocytes are known for slow propagation and their inability to form a suitable monolayer in 2D culture [51]. This correlates with a decrease in tight junctions alongside the peribiliary matrix remodelling and ductopenia seen in vivo [50]. Using TEER techniques, Tabibian et al. [51] were able to monitor the tight junctions within cholangiocyte cultures, critical to the study of PSC pathology. 

### 4.2. Monitoring Hepatic Tight Junctions in Models of Cholestasis

Chlorpromazine (CPZ), an antipsychotic drug, is associated with idiosyncratic toxicity and causing intrahepatic cholestasis in a short period of time. The precise mechanism is unknown, and some patients can tolerate the drug with no hepatotoxic side effects. Cholestasis is characterized by impaired ability of the bile ducts to secrete bile acids, bilirubin and cholesterol [52] and CPZ has long been used as a model drug to induce cholestasis in vitro [53,54,55,56].

As there is currently a need within the pharmaceutical industry to predict cholestasis in vitro before pre-clinical human trials, we have used the HepaRG^TM^ cell line, with its intrinsic co-culture of hepatocytes and cholangiocytes, to attempt to further elucidate the effect of CPZ on both cell populations [40]. Understanding the mechanisms behind CPZ toxicity may also provide insight into studies of other xenobiotics that are also known to target the biliary system/cholangiocytes and aid in pre-clinical toxicity studies. 

**Figure 5:**
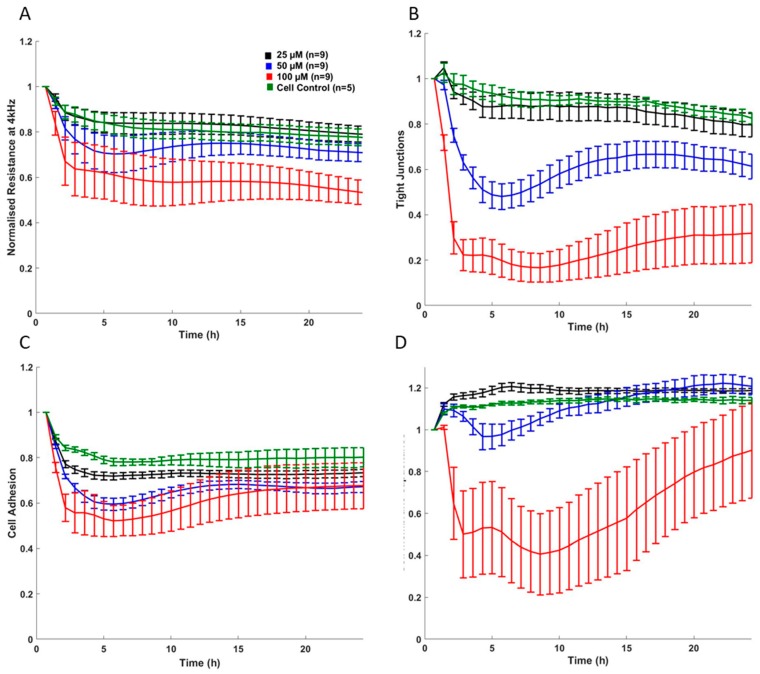
Impedance signature showing an example of cholestasis induced by chlorpromazine. (**A**) Post-challenge impedance kinetics showing 24 h treatment with CPZ in confluent HepaRG cells; CPZ caused a dose-dependent decline in normalized resistance—a global indicator of cellular status. (**B**) Rb (cell–cell tight junctions); CPZ disrupted tight junctions in a concentration- and time-dependent manner, compared with the control values. Subjecting HepaRG to 100 μM CPZ caused Rb to decrease after 6 h, with marginal recovery around 15 h. (**C**) α (basolateral adhesion); cell-substrate adhesion disruption was detected in a concentration-dependent manner, suggesting some loosening of cells from the electrode surface. At 100 μM CPZ, cell adhesiveness decreased, but showed an increase around 10 h, suggesting re-adherence of cells. (**D**) Cm (membrane capacitance); membrane capacitance, reflecting cell membrane integrity, was significantly compromised at 100 μM CPZ, beginning at 2 h post treatment. Some increase in Cm at 100 μM CPZ was seen at around 10 h, which is consistent with the basolateral adhesion (α) and further indicating some cellular compensatory effect. (Figure reproduced from Morgan et al., 2019) [40].

Our data showed that CPZ causes alterations to cell–cell junctions, adhesion and total membrane integrity [40]. A sharp drop in deconvoluted impedance data showed that cell membrane integrity and tight junctions decreased in the first 6 hours of CPZ exposure [40]. Further investigation using qRTPCR showed that the bile acid transporter ABCB11 was downregulated, leading us to theorize that the accumulation of bile acids causes a loss of tight junctions, leading to hepatic injury and cholestasis [40] (Figure 5).

## 5. Hepatocellular Cancer 

The growth kinetics of cancer cells are based on several factors; acceleration of cell-cycle, percentage of proliferating cells within a tumour, a quantitative measurement of cell senescence and number of cells lost by other means. Using these factors, clinically valuable information can be applied to treatment regimens; for example, the proliferation rate of a specific tumour can be used to develop a specific course of radiotherapy. Morphologically, there can be changes in shape and size of the nucleus, where nucleoli are easily seen and cytoplasm is scarce [57]. Cytoskeletal changes and proliferation can be detected using IBCAs, and there are a number of studies which discuss using impedance to detect the changes in morphology, growth kinetics, and the proliferation of cancer cells [50,58,59].

### 5.1. Monitoring of Migration and Proliferation of Hepatocellular Cancer Cells

The mechanism of liver cancer metastasis is not fully understood. Insight into cell migration and the proliferation of cancerous hepatocytes could aid in the development of new drug therapies targeting metastasis. Saxena et al. [60] used ECIS Zθ to monitor co-cultures of HepG2 or Huh7 cells with human umbilical vascular endothelial cells (HUVECs) to study the effects of leptin on hepatocellular carcinoma (HCC) [60]. ECIS arrays were seeded with HUVECs and spreading monitored for 2.5 h. After 2.5 h, a level of confluency was reached, which gave a stable impedance signal [60]. The introduction of Huh-7 or HepG2 cells to the culture at this point produced a marked decrease in impedance suggestive of the invasive properties of these cell lines. Impedance was further decreased when leptin treated cells were used, suggesting increased invasive behaviour in the presence of leptin [60].

Wang et al. [61] have demonstrated the application of ECIS in the real-time analysis of cell cycle in HeLa cells. This is possible due to changes in surface morphology and adhesive properties that occur in cells in response to cell cycle [62]. As cancer cells grow and divide without check points, Wang et al. [61] developed a bio-electronic chip system to continuously monitor the morphological changes related to cell cycle using impedance. The cell division was synchronised by thymidine at various stages and the impedance modelling of the cell cycle, validated by flow cytometry, showed a clear correlation of impedance curves with cell synchronicity.

While this was the first study of its kind to correlate impedance and flow cytometry, there is potential to study cell cycling in HCC.

### 5.2. Role of Cellular Impedance as a Diagnostic System for HCC

While we have focused mainly on IBCA and its uses in hepatology research, it is important to remember that other impedance techniques can be used in acellular biochemical assays.

Impedance measurement has been applied to immunochemical diagnosis of HCC using specific antibodies and patient serum similar to other end-point antibody assays such as ELISAs but giving immediate high throughput results which are clinically relevant.

HCC has a high mortality rate due to the difficulty of early detection and diagnosis [63]. Better biomarkers and new techniques to detect HCC earlier are important for saving millions of lives per year [63]. Chen et al. [63] have devised an EIS system that exploits levels of human cervical cancer oncoprotein-1 (HCCR-1) which has been shown to have a 95.7% specificity to HCC enabling detection at an early stage. In their study, instead of culturing cells on an electrode, a gold electrode surface was modified to maintain a self-assembly monolayer using a calixarene-based ProLinker B [63]. Calixarenes are cup-shaped molecules which offer a space for a molecule or antibody to bind. The electrode coated with ProLinker B was incubated with HCCR-1 antibody to which it binds by the Fc domain, leaving the specific HCCR-1 binding sites exposed [63]. Patient serum was then introduced. If the serum contained HCCR-1, it bound itself to the exposed site, altering the impedance signal [63]. The difference in signal between that of the empty electrode and the electrode with ProLinker B plus antibody gave a specific impedance signal for HCC-1 positive serum [63]. Using EIS and optimising dilution factors of serum, a ratio was developed distinguishing between healthy subjects and subjects testing positive for HCC.

This simple and non-invasive technique to directly detect early stage HCC could also be useful in other cancer research, where specific antibodies can offer early detection with a high degree of accuracy. Once a specific marker of a disease state has been identified, specific antibody- and impedance-based technology can be applied for high-throughput and rapid diagnostic testing. This is especially advantageous where early diagnosis leads to earlier intervention and lower mortality.

## 6. Mechanistic Impedance Signatures

Mechanistic impedance signatures measure intrinsic or structural changes in cell culture caused by the addition of a drug or compound. This can range between monitoring changes in cellular attachment to drug-induced changes of membrane receptors or the changes caused by a drug to the cytoskeleton.

It has been shown that monitoring drugs with different mechanisms of action show different impedance profiles [14]. The ability to characterise a specific mechanism of action of a drug using IBCAs would be beneficial to the pharmaceutical industry in providing high-throughput, non-invasive assessment of new compounds and therapeutics. IBCAs have proven useful in producing specific impedance signatures based on the mechanism of action of various compounds [64]. For instance, Kustermann et al. [14] differentiated between cytotoxic and cytostatic drugs using xCELLigence™. The specific signatures of calcium modulators, antimitotic and DNA-damaging agents were assessed by Atienzar et al. [42] and while they found an impedance trend when monitoring the drugs responsible for a particular mechanism of action, they stated that cell imaging alone is not optimal and a combination of reliable analysis tools are needed to standardize these types of results. Therefore, it may be possible to reliably predict a mechanism of action based on impedance data that is supported by biochemical assays and classical viability assays. However, a standardized approach is needed in this area and it may be prudent to produce guidelines similar to the minimum information for publication of quantitative real-time PCR experiments (MIQE guidelines) currently used in publishing gene expression data to create a model that ensures stringent and accurate reporting [65]. 

## 7. Cellular Impedance in Understanding the Gut/Liver Axis

Increasing importance has been placed on the interaction between the gut and the liver in the context of the development of liver disease. As this field is rapidly evolving it is becoming increasingly clear that many liver diseases such as alcoholic liver disease, non-alcoholic fatty liver disease and cholestatic syndromes may originate in the gut [66,67].

Many impedance-based studies monitoring the gut–liver axis have used TEER when assessing the barrier function of intestinal cells, usually CACO2s (ATCC^®^ HTB-37™), and their relation to the enterohepatic circulation [68]. The gut plays a crucial role in the influx of drugs, metabolites and toxins to the liver. Therefore, the monitoring of gut barrier function can be important when assessing the effects of a compound on first pass metabolism and even on repeat dosing as it flows through the gut and repeatedly into the liver, where molecules often undergo transformation into toxic metabolites before conjugation.

Lee and Sung [69] have produced a model mimicking the absorption of fatty acids to assess the interaction between the two tissues, allowing assessment of the multifactorial systems involved in hepatic steatosis, or fatty liver disease. There are many components to hepatic steatosis and the mechanisms of disease are still not fully understood. By studying the various impedance signatures of the gut and thee liver, and how each cell type responds to challenge with drugs or dietary components, we can gain a clearer picture of what happens in real time. Ability to do this in transwell systems overcomes the limitation of conventional mono-cultures and could provide a unique insight to the interactions and possible cross-talk between these two organs. Lee and Sung [69] have gained insight into the role the gut barrier function plays in hepatic steatosis by employing a TEER transwell system which mimics the systemic circulation in vivo. They found that the tightening of the tight junctions of the gut in response to certain compounds significantly reduced lipid accumulation, whereas when junctions were compromised using inflammatory factors lipid accumulation increased [69]. Parallel to these findings, adding these compounds or inflammatory factors directly to hepatocytes had no effect on lipid accumulation [69]. Further work on the gut–liver axis using an impedance-based methodology is not only relevant, but could provide unique insights into the mechanisms of disease and aid in finding novel therapeutics.

## 8. Novel Development in Cellular Impedance

### D Platforms and Microfluidic Systems

The ability to assess biological phenomena within 3D structures would create a more relevant model for pre-clinical toxicity screening. With recent advances in microfluidics, bio-printing and hydrogel-based methods, researchers have been able to develop new techniques for fabricating and probing in vitro biological systems for use as a more relevant cell culture model. Combining 3D in vitro human models with impedance based cellular assays will provide a platform for a non-invasive, quantitative, detailed analysis of cell culture in real time, hugely benefiting the in vitro drug screening process and making animal testing less attractive. Recent developments in microelectrode sensor design for electrical impedance tomography show great promise in their translation to the field of tissue engineering and 3D culture [70,71,72].

Microfluidic devices have been popular in providing ‘organ-on-a-chip’ technology that can mimic circulation within an organism, hence their huge potential in toxicity studies.

Tran et al. [73] created a device combining ECIS technology with a high-throughput microfluidic network in a hydrogel-based diffusion chip. This was used to estimate the IC50 and monitor the toxic gradient of doxorubicin and 5-fluororacil on HeLa cervical cancer cells and NIH-3T3 murine embryonic fibroblast cells [73]. Their microfluidic device consisted of three modules; micro-fluidic vessels, a hydrogel chamber to replicate tissue structure and ensure a gradient of solution and an ECIS microelectrode component to monitor cell viability when cells were exposed to the toxic gradient [73]. They then compared the impedance-based measurements with an MTS colorimetric assay of cell viability in standard culture [73]. Using the high-throughput micro-fluidic device, they successfully predicted the IC50 values for doxorubicin and 5-fluororacil [73].

The fabrication of bespoke IBC arrays having specific numbers/sizes of electrodes increases the flexibility of IBCA for real time monitoring of cells in vitro. As previously stated, various array designs are available for use in 2D culture, depending on experimental needs. As cellular impedance can only be monitored on cells grown directly on the electrode, large electrodes increase the sampling size and reduce variability. Smaller electrodes ensure that the total current passes through the cell monolayers and the application of a range of AC frequencies makes it possible to optimise impedance for various parameters (Rb, α, Cm) by the deconvolution of data. However, measuring impedance in 3D scaffold models or in spheroids still has limitations, due to the placement and size of electrodes within cultures.

Hiber et al. [74] used different-sized electrodes to monitor changes in impedance of a 3D HepaRG cell culture grown on scaffolds and incubated with various concentrations of APAP. They used larger parallel plate electrodes combined with small needle like photo-lithographically structured electrodes which were spaced at the sub-mm level. They concluded that smaller electrodes, sampling a more restricted area, were more sensitive and better able to capture the dielectric properties of the cell culture than the larger electrodes which detected more background noise of the current as it moved through the media.

## 9. Conclusions 

In conclusion, cellular impedance assays have an important role in understanding the pathophysiology of liver disease, cancer diagnostics, viral infection and drug development.

The major advantage of this technique is real-time data collection in a label-free manner; therefore, studies can be extended over a period of time necessary to investigate the effect of a compound or treatment for as long as required. It is particularly advantageous in hepatology, due to its ability to monitor changes in tight junctions, a known point of liver pathologies.

The application of cellular impedance to hepatology in combination with current techniques offers the opportunity to advance the study and diagnosis of liver disease and facilitate drug development. In addition, it complies with the 3Rs and is invaluable in reducing, refining and replacing animals. In both areas it offers the possibility of developing HTS and reducing costs. This would be especially valuable in drug testing for the early exclusion of compounds from further expensive clinical trial testing.

## References

[1] Randivir E.P., Banks C.E. (2013). Electrochemical impedance spectroscopy: An overview of bioanalytical applications. Anal. Methods.

[2] Gamal W., Treskes P., Samuel K., Sullivan G., Siller R., Srsen V., Morgan K., Bryans A., Kozlowska A., Underwood I. (2017). Low-dose acetaminophen induces early disruption of cell-cell tight junctions in human hepatic cells and mouse liver. Sci. Rep..

[3] García-Sánchez T., Bragós R., Mir L.M. (2018). In vitro analysis of various cell lines responses to electroporative electric pulses by means of electrical impedance spectroscopy. Biosens. Bioelectron..

[4] Bagnaninchi P.O., Drummond N. (2011). Real-time label-free monitoring of adipose-derived stem cell differentiation with electric cell-substrate impedance sensing. Proc. Natl. Acad. Sci. USA.

[5] Rammah M., Dandachi F., Salman R., Shihadeh A., El-Sabban M. (2012). In vitro cytotoxicity and mutagenicity of mainstream waterpipe smoke and its functional consequences on alveolar type II derived cells. Toxicol. Lett..

[6] Moodley K., Angel C.E., Glass M., Graham E.S. (2011). Real-time profiling of NK cell killing of human astrocytes using xCELLigence technology. J. Neurosci. Methods.

[7] Sergent J.A., Paget V., Chevillar S. (2012). Toxicity and genotoxicity of nano-SiO_2_ on human epithelial intestinal HT-29 cell line. Ann. Occup. Hyg..

[8] Diemert S., Dolga A.M., Tobaben S., Grohm J., Pfeifer S., Oexler E., Culmsee C. (2012). Impedance measurement for real time detection of neuronal cell death. J. Neurosci. Methods.

[9] Yang T., Mei H., Xu D., Zhou W., Zhu X., Sun L., Huang X., Wang X., Shu T., Liu J. (2017). Early indications of ANIT-induced cholestatic liver injury: Alteration of hepatocyte polarization and bile acid homeostasis. Food Chem. Toxicol..

[10] Michaelis S., Wegener J., Robelek R. (2013). Label-free monitoring of cell-based assays: Combining impedance analysis with SPR for multiparametric cell profiling. Biosens. Bioelectron..

[11] Giaever I., Keese C.R. (1984). Monitoring Fibroblast Behaviour in Tissue Culture with an Applied Electric Field. Proc. Natl. Acad. Sci. USA.

[12] Tibaldi L., Leyman S., Nicolas A., Notebaert S., Dewulf M., Ngo T.H., Zuany-Amorim C., Amzallag N., Bernard-Pierrot I., Sastre-Garau X. (2013). New blocking antibodies impede adhesion, migration and survival of ovarian cancer cells, highlighting MFGE8 as a potential therapeutic target of human ovarian carcinoma. PLoS ONE.

[13] Sansing H., Renner N., MacLean A.G. (2012). An inverted blood-brain barrier model that permits interactions between glia and inflammatory stimuli. J. Neurosci. Methods.

[14] Kustermann S., Boess F., Buness A., Schmitz M., Watzele M., Weiser T., Singer T., Suter L., Roth A. (2013). A label-free impedance-based real time assay to identify drug-induced toxicities and differentiate cytostatic from cytotoxic effects. Toxicol. In Vitro.

[15] Marinova Z., Waltiza S., Grunblatt E. (2013). 5-HT2A serotonin receptor agonist DOI alleviates cytotoxicity in neuroblastoma cells: Role of the ERK pathway. Prog. Neuro Psychopharmacol. Biol. Psychiatry.

[16] Moniri M.R., Young A., Reinheimer K., Rayat J., Dai L.J., Warnock G.L. (2015). Dynamic assessment of cell viability, proliferation and migration using real time cell analyser system (RTCA). Cytotechnology.

[17] Benson K., Cramer S., Galla H.J. (2013). Impedance-Based Cell Monitoring: Barrier Properties and Beyond. Fluids Barriers CNS.

[18] Srinivasan B., Kolli A.R., Esch M.B., Abaci H.E., Shuler M.L., Hickman J.J. (2015). TEER Measurement Techniques for In Vitro Barrier Model Systems. J. Lab. Autom..

[19] Lee N.P., Luk J.M. (2010). Hepatic tight junctions: From viral entry to cancer metastasis. World J. Gastroenterol..

[20] Mitic L., Anderson J.M. (1998). Molecular architecture of tight junctions. Annu. Rev. Physiol..

[21] Claude P., Goodenough D.A. (1973). Fracture faces of zonulae occludentes from ‘tight’ and ‘leaky’ epithelia. J. Cell Biol..

[22] Kojima T., Yamamoto T., Murata M., Chiba H., Kokai Y., Swada N. (2003). Regulation of the blood-biliary barrier: Interaction between gap and tight junctions in hepatocytes. Med. Electron. Microsci..

[23] Nathanson M.H., Rios-Velez L., Burgstahler A.D., Mennone A. (1999). Communication via gap junctions modulates bile secretion in the isolated perfused rat liver. Gastroenterology.

[24] Gitlin N., Arias I.M., Boyer J.L., Chisari F.V., Fausto N., Schachter D., Schafritz D.A. (2001). The Liver Biology and Pathobiology.

[25] Zeisel M.B., Dhawan P., Baumert T.F. (2019). Tight junction proteins in gastrointestinal and liver disease. Gut.

[26] Lódi C., Szabó E., Holczbauer A., Batmunkh E., Szíjártó A., Kupcsulik P., Kovalszky I., Paku S., Illyés G., Kiss A. (2006). Claudin-4 differentiates biliary tract cancers from hepatocellular carcinomas. Mod. Pathol..

[27] Benedicto I., Molina-Jimenez F., Bartosch B., Cosset F.L., Lavilette D., Prieto J., Moreno-Otero R., Valenzuela-Fernandez A., Aldabe R., Lopez-Cabrera M. (2009). The tight junction-associated protein occludin is required for a postbinding step in hepatitis C virus entry and infection. J. Virol..

[28] Mankouri J., Walter C., Stewart H., Bentham M., Park W.S., Do Heo W., Fukuda M., Griffin S., Harris M. (2016). Release of Infectious Hepatitis C Virus from Huh7 Cells Occurs via a trans-Golgi Network-to-Endosome Pathway Independent of Very-Low-Density Lipoprotein Secretion. J. Virol..

[29] Teimourpour R., Meshkat Z., Gholoubi A., Nomani H., Rostami S. (2015). Viral Load Analysis of Hepatitis C Virus in Huh7.5 Cell Culture System. Jundishapur J. Microbiol..

[30] Sainz B., Barretto N., Uprichard S.L. (2009). Hepatitis C virus infection in phenotypically distinct Huh7 cell lines. PLoS ONE.

[31] Bartenschlager R., Pietschmann T. (2005). Efficient hepatitis C virus cell culture system: What a difference the host cell makes. Proc. Natl. Acad. Sci. USA.

[32] Ndongo-Thiam N., Berthillon P., Errazuriz E., Bordes I., De Sequeira S., Trépo C., Petit M.A. (2011). Long-term propagation of serum hepatitis C virus (HCV) with production of enveloped HCV particles in human HepaRG hepatocytes. Hepatology.

[33] Fletcher N.F., Clark A.R., Balfe P., McKeating J.A. (2017). TNF superfamily members promote hepatitis C virus entry via an NF-κB and myosin light chain kinase dependent pathway. J. Gen. Virol..

[34] Yoneda M., Hyun J., Jakubski S., Saito S., Nakajima A., Schiff E.R., Thomas E. (2016). Hepatitis B Virus and DNA Stimulation Trigger a Rapid Innate Immune Response through NF-κB. J. Immunol..

[35] Kasai F., Hirayama N., Ozawa M., Satoh M., Kohara A. (2018). HuH-7 reference genome profile: Complex karyotype composed of massive loss of heterozygosity. Hum. Cell.

[36] Nakabayashi H., Miyano K., Sato J., Yamane T., Taketa K. (1982). Growth of human hepatoma cell lines with differentiated functions in chemically defined medium. Cancer Res..

[37] Castell J.V., Gomez-Lechon Maria J., Ponsoda X., Bort R. (1996). In vitro investigation of the molecular mechanisms of hepatotoxicity. In Vitro Methods in Pharmaceutical Research.

[38] Gerets H.H., Tilmant K., Gerin B., Chanteux H., Depelchin B.O., Dhalluin S., Atienzar F.A. (2012). Characterization of primary human hepatocytes, HepG2 cells, and HepaRG cells at the mRNA level and CYP activity in response to inducers and their predictivity for the detection of human hepatotoxins. Cell Biol. Toxicol..

[39] Pennington M.R.R., Van de Walle G.R. (2017). Electric cell-substrate impedance sensing to monitor viral growth and study cellular responses to infection with alphaherpesviruses in real time. mSphere.

[40] Morgan F., Martucci N., Kozlowska A., Gamal W., Brzeszczynki F., Treskes P., Samuel K., Hayes P., Nelson L., Bagnaninchi P. (2019). Chlorpromazine toxicity is associated with disruption of cell membrane integrity and initiation of a pro-inflammatory response in the HepaRG hepatic cell line. Biomed. Pharmacother..

[41] Atienzar F.A., Tilmant K., Gerets H.H., Toussaint G., Speeckaert S., Hanon E., Depelchin O., Dhalluin S. (2011). The use of real-time cell analyser technology in drug discovery: Defining optimal cell culture conditions and assay reproducibility with different adherent cellular models. J. Biomol. Screen.

[42] Atienzar F.A., Gerets H., Tilmant K., Toussain G., Dhalluin S. (2013). Evaluation of impedance-based label-free technology as a tool for pharmacology and toxicology investigations. Biosensors.

[43] Peyre L., de Sousa G., Barcellini-Couget S., Luzy A.P., Zucchini-Pascal N., Rahmini R. (2015). High-content screening imaging and real-time cellular impedance monitoring for the assessment of chemical’s bio-activation with regards hepatotoxicity. Toxicol. In Vitro.

[44] Gripon P., Rumin S., Urban S., Le Seyec J., Glaise D., Cannie I., Guyomard C., Lucas J., Christian T., Guguen-Guillouzo C. (2002). Infection of a human hepatoma cell line by hepatitis B virus. Proc. Natl. Acad. Sci. USA.

[45] Le Vee M., Jouan E., Stieger B., Fardel O. (2013). Differential regulation of drug transporter expression by all-trans retinoic acid in hepatoma HepaRG cells and human hepatocytes. Eur. J. Pharm. Sci..

[46] Szabo M., Veres Z., Baranyai Z., Jakab F., Jemnitz K. (2013). Comparison of Human Hepatoma HepaRG cells with Human and Rat Hepatocytes in Uptake Transport Assays in Order to Predict a Risk of Drug Induced Hepatotoxicity. PLoS ONE.

[47] Chun Y.S., Chaudhari P., Jan Y.Y. (2010). Applications of Patient-Specific Induced Pluripotent Stem Cells; Focused on Disease Modeling, Drug Screening and Therapeutic Potentials for Liver Disease. Int. J. Biol. Sci..

[48] Sullivan G.J., Hay D.C., Park I.H., Fletcher J., Hannoun Z., Payne C.M., Dalgetty D., Black J.R., Ross J.A., Samuel K. (2010). Generation of Functional Human Hepatic Endoderm from Human Induced Pluripotent Stem Cells. Hepatology.

[49] Rashid S.T.T., Alexander G.J.M.J. (2013). Induced pluripotent stem cells: From Nobel Prizes to clinical applications. J. Hepatol..

[50] Zhou Y., Yang D., Zhou Y., Khoo B.L., Han J., Ai Y. (2017). Characterizing Deformability and Electrical Impedance of Cancer Cells in a Microfluidic Device. Anal. Chem..

[51] Tabibian J.H., Trussoni C.E., O’hara S.P., Splinter P.L., Heimbach J.K., LaRusso N.F. (2014). Characterization of cultured cholangiocytes isolated from livers of patients with primary sclerosing cholangitis. Lab. Investig..

[52] Anthérieu S., Azzi P.B., Dumont J., Abdel-Razzak Z., Guguen-Guillouzo C., Fromenty B., Robin M.A., Guillouzo A. (2013). Oxidative stress plays a major role in chlorpromazine-induced cholestasis in human HepaRG cells. Hepatology.

[53] Anthérieu S., Chesné C., Li R., Guguen-Guillouzo C., Guillouzo A. (2012). Optimization of the HepaRG cell model for drug metabolism and toxicity studies. Toxicol. In Vitro.

[54] Akerboom T., Schnieder I., vom Dahl S., Sies H. (1991). Cholestasis and changes of portal pressure caused by chlorpromazine in the perfused rat liver. Hepatology.

[55] Zimmerman H.J., Lewis J.H. (1987). Drug-induced cholestasis. Med. Toxicol..

[56] Moradpour D., Altorfer J., Flury R., Greminger P., Meyenberger C., Schmid M. (1994). Chlorpromazine-induced vanishing bile duct syndrome leading to biliary cirrhosis. Hepatology.

[57] Baba A.I., Câtoi C. (2007). Comparative Oncology.

[58] Zou Y., Guo Z. (2003). A review of electrical impedance techniques for breast cancer detection. Med. Eng. Phys..

[59] Hong J., Kandasamy K., Marimuthu M., Choi C.S., Kim S. (2011). Electrical cell-substrate impedance sensing as a non-invasive tool for cancer cell study. Analyst.

[60] Saxena N.K., Sharma D., Ding X., Lin S., Marra F., Merlin D., Anania F.A. (2007). Concomitant activation of the JAK/STAT, PI3K/AKT, and ERK signaling is involved in leptin-mediated promotion of invasion and migration of hepatocellular carcinoma cells. Cancer Res..

[61] Wang L., Yin H., Xing W., Yu Z., Guo M., Cheng J. (2010). Real-time, label-free monitoring of the cell cycle with a cellular impedance sensing chip. Biosens. Bioelectron..

[62] Porter K., Prescott D., Frye J. (1973). Changes in surface morphology of Chinese hamster ovary cells during the cell cycle. J. Cell Biol..

[63] Chen D., Shen M., Cao Y., Bo B., Chen Z., Shu Y., Li G. (2013). Electrochemical identification of hepatocellular carcinoma based on the assay of human cervical cancer oncoprotein-1 in serum. Electrochem. Commun..

[64] Abassi Y.A., Xi B., Zhang W., Ye P., Kirstein S.L., Gaylord M.R., Feinstein S.C., Wang X., Xu X. (2009). Kinetic cell-based morphological screening: Prediction of mechanism of compound action and off-targets effects. Chem. Biol..

[65] Bustin S.A., Benes V., Garson J.A., Hellemans J., Huggett J., Kubista M., Mueller R., Nolan T., Pfaffl M.W., Shipley G.L. (2009). The MIQE guidelines: Minimum information for publication of quantitative real-time PCR experiments. Clin. Chem..

[66] Szabo G. (2015). Gut-Liver axis in alcoholic liver disease. Gastroenterology.

[67] Weist R., Abillos A., Trauner M., Bajaj J.S., Jilan R. (2017). Targeting the gut-liver axis in liver disease. J. Hepatol..

[68] Tan H.-Y., Trier S., Rahbek U.L., Dufva M., Kutter J.P., Andresen T.L. (2018). A multi-chamber microfluidic intestinal barrier model using Caco-2 cells for drug transport studies. PLoS ONE.

[69] Lee S.Y., Sung J.H. (2018). Gut-liver on a chip toward an in vitro model of hepatic steatosis. Biotechnol. Bioeng..

[70] Wu H., Yang Y., Bagnaninchi P., Jia J. (2018). Electrical impedance tomography for real-time and label-free cellular viability assays of 3D tumour spheroids. Analyst.

[71] Wu H., Zhou W., Yang Y., Jia J. (2018). Exploring the Potential of Electrical Impedance Tomography for Tissue Engineering Applications. Materials.

[72] Yang Y., Jia J., Smith S., Jamil N., Gamal W., Bagnaninchi P.O. (2017). A Miniature Electrical Impedance Tomography Sensor and 3-D Image Reconstruction for Cell Imaging. IEEE Sens. J..

[73] Tran T.B., Cho S., Min J. (2013). Hydrogel-based diffusion chip with Electric Cell-substrate Impedance Sensing (ECIS) integration for cell viability assay and drug toxicity screening. Biosens. Bioelectron..

[74] Hilber W., Lornejad-Schäfer M.R., Schäfer C., Lederer T., Schröder K., Jakoby B. (2011). Impedance Spectroscopy of a Human Hepatic 3D Cell Model in-Vitro: A Comparative Study with Differently Shaped Electrodes. Procedia Eng..

